# Influenza A Viruses in the Swine Population: Ecology and Geographical Distribution

**DOI:** 10.3390/v16111728

**Published:** 2024-11-01

**Authors:** Nailya Klivleyeva, Nurbol Saktaganov, Tatyana Glebova, Galina Lukmanova, Nuray Ongarbayeva, Richard Webby

**Affiliations:** 1 The Research and Production Center for Microbiology and Virology, Almaty 050010, Kazakhstan; i_nailya@list.ru (N.K.); nsaktaganov1984@mail.ru (N.S.); gal_l@bk.ru (G.L.); nuray.syrlybay@gmail.com (N.O.); 2 Department of Infectious Disease, St. Jude Children’s Research Hospital, Memphis, TN 38105-3678, USA; richard.webby@stjude.org

**Keywords:** swine influenza, reassortment, epizootiology, zoonotic infection, transmission

## Abstract

Despite the efforts of practical medicine and virology, influenza viruses remain the most important pathogens affecting human and animal health. Swine are exposed to infection with all types of influenza A, B, C, and D viruses. Influenza viruses have low pathogenicity for swine, but in the case of co-infection with other pathogens, the outcome can be much more serious, even fatal. Having a high zoonotic potential, swine play an important role in the ecology and spread of influenza to humans. In this study, we review the state of the scientific literature on the zoonotic spread of swine influenza A viruses among humans, their circulation in swine populations worldwide, reverse zoonosis from humans to swine, and their role in interspecies transmission. The analysis covers a long period to trace the ecology and evolutionary history of influenza A viruses in swine. The following databases were used to search the literature: Scopus, Web of Science, Google Scholar, and PubMed. In this review, 314 papers are considered: n = 107 from Asia, n = 93 from the U.S., n = 86 from Europe, n = 20 from Africa, and n = 8 from Australia. According to the date of publication, they are conditionally divided into three groups: contemporary, released from 2011 to the present (n = 121); 2000–2010 (n = 108); and 1919–1999 (n = 85).

## 1. Introduction

**Figure 1:**
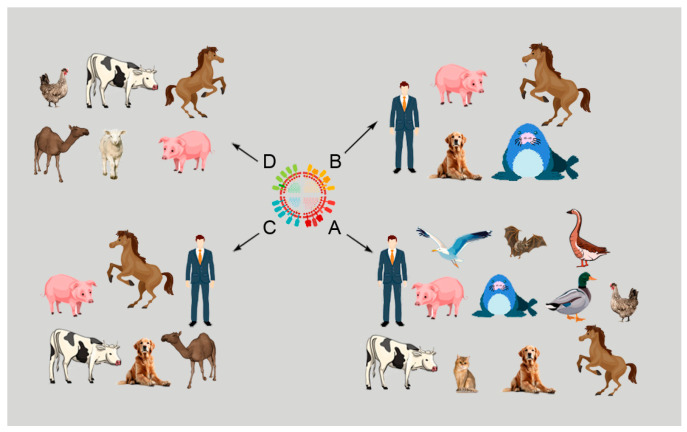
Influenza A, B, C, and D in different species of mammals.

Influenza viruses (IVs) belong to the *Orthomyxoviridae* family and have a segmented genome with single-stranded, negative-sense RNA. Based on genetic and antigenic differences, IVs are divided into four genera, A, B, C, and D, which infect different species of mammals and birds with the four types of IV that occur in swine [1,2,3,4]. Among the four types of IVs, influenza A viruses (IAVs) are highly significant pathogens responsible for serious epidemics in humans and domestic animals. Figure 1 presents different species of animals infected with the four types of IVs.

The IAV genome consists of eight RNA segments: PB2, PB1, PA, hemagglutinin (HA), NP, neuraminidase (NA), M, and NS [1]. Two surface antigens, HA and NA, are mainly subject to antigenic variability, while internal proteins (NP and M) are relatively conservative. HA and NA play an essential role in the initial stages of cell infection [5]. HA binds to host cell receptors that contain terminal sialic acid residues (-2,6-SA or -2,3-SA). The HA gene is in charge of attaching viral pieces to the host cell receptor. NA removes the cell surface receptor (sialic acid); this is crucial for releasing viral particles from the cell surface and virus spillover [5].

**Figure 2:**
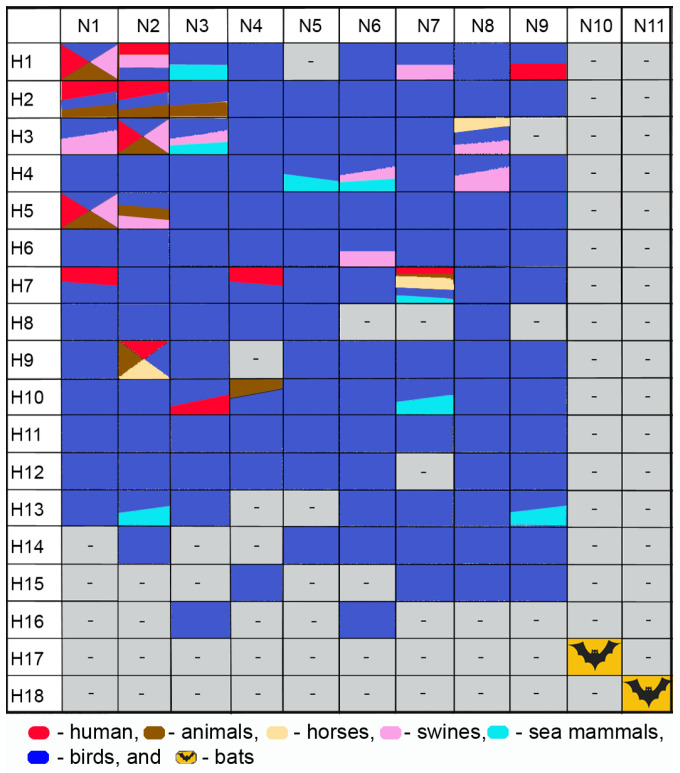
Influenza subtypes of viruses H1–H18 and N1–N11 in different species of mammals.

Based on major antigenic differences in surface proteins (HA and NA), 18 HA subtypes (H1–H18) and 11 NA subtypes (N1–N11) have been identified. The H1–H16 and N1–N9 virus subtypes have been identified in waterfowl, which are believed to constitute the natural IAV reservoir [6,7,8], while the H17–H18 and N10–N11 subtype sequences have been found in bats [9,10,11]. Only a limited number of subtypes have been found in mammals, such as H1 and H3 viral subtypes, which we identified and circulate in both humans and swine [12,13,14,15,16,17,18,19]. Figure 2 presents subtypes of H1–H18 hemagglutinins and N1–N11 neuraminidases of influenza viruses in different species of mammals.

IAVs’ epidemiology and ecology are complicated by their multi-host and segmented genome. IAVs are among the main pathogens in swine that give rise to acute respiratory infections and cause significant economic losses for swine farms. In addition to swine IAVs, human and avian IAVs can also infect swine. Swine are believed to serve as an intermediate “mixing vessel” for generating a variety of novel IVs [20]. The co-infection of swine with two or more IAV strains can induce reassortment [21,22], which in turn can promote the emergence of new IV strains [23,24,25]. There are numerous reports of zoonotic infections in humans with swine IAVs and reverse zoonotic infections in swine with human IAVs [26,27,28,29,30].

A literature search on the review topic was conducted on knowledge-intensive databases, including Scopus, Web of Science, Google Scholar, and PubMed. The following words and phrases were used as criteria for the literature search: “influenza viruses”, “classification and structure of the influenza virus”, “swine flu”; “avian flu viruses”, “reassortment”; “epizootology”; “zoonotic infection”; “transmission”, “swine flu in humans”, and “swine flu in horses”.

The following sections of this review assess swine influenza A viruses, their ecology, and their geographic distribution.

## 2. Ecology of Influenza Virus

Due to recurrent outbreaks and fast spillover, differing both genetically and antigenically, IAVs pose a serious problem for both swine production and public health. This issue is considered not only from the point of view of estimating and controlling the presently developing strains in swine but also in terms of describing their zoonotic potential [31].

IAVs infect various animal species, and swine (*Sus scrofa*) are among the natural hosts for these viruses. Swine influenza (SI) is an extremely dangerous respiratory disease in swine. The major causative viruses include the H1N1, H1N2, and H3N2 subtypes [32], which are antigenically related to human IV.

The development of IAV proceeds very rapidly due to two key mechanisms: antigenic drift and antigenic shift. Antigenic drift occurs due to the gradual accumulation of mutations in the surface proteins HA and NA, which leads to antigenic changes in IAV. Antigenic drift is responsible for human seasonal influenza viruses. Antigenic shift, otherwise known as reassortment, happens due to the segmented IV genome. When two or more different IAVs infect a cell or a host, the viral gene segments can be intermixed and reassorted, leading to the appearance of different reassortant viruses. Examples of antigenic shift include the emergence of three of the four pandemic IVs that have occurred in human history, which caused the 1957 H2N2 Asian flu pandemic, the 1968 Hong Kong H3N2 flu pandemic, and the 2009 H1N1 flu pandemic [33,34].

It was discovered that swine tissues express both α-2,6-SA (human IAV receptor preference) and α-2,3-SA (avian IAV receptor preference) [20]. Genetic reassortment between human and/or avian and/or swine IAVs can take place in swine. A prime example of this reassortment is the 2009 influenza pandemic caused by the H1N1 swine influenza reassortant virus containing the NA and M genes from the Eurasian swine virus and six remaining genes from the North American triple-reassortant virus [35,36]. The possibility of creating novel IAVs has led to swine becoming known as “mixing vessels” [24,34,37]. However, whether swine are necessary intermediate hosts for bird-to-human infections is a debatable matter because people and certain land birds have also been found to express both types of sialic acids [38,39,40]. At the same time, swine play a major role in IAV ecology, and this is especially true of human–swine interactions. Infection in swine can nevertheless cause viruses to acquire the adaptation needed either to maintain infection inside swine, thereby ensuring an additional reservoir for human infection, or to provide the mutations necessary for transmission to humans, which will lead to the establishment of that virus in the human population [27,41,42,43].

## 3. Geographical Distribution of Swine Influenza Viruses

**Figure 3:**
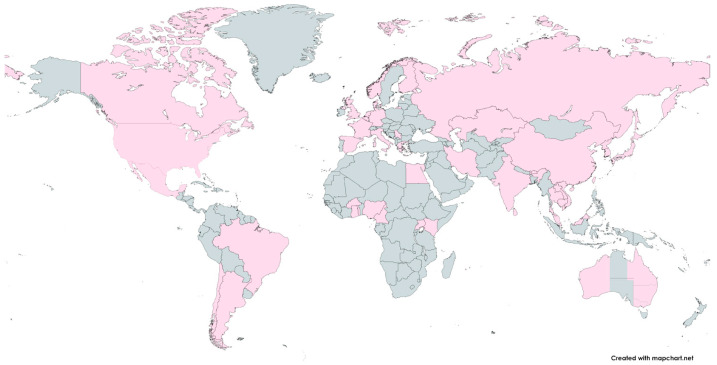
The geographical distribution of swine influenza viruses. The locations where influenza viruses were detected in pigs are marked in pink. The world map was created online at https://mapchart.net (accessed on 11 October 2024).

There is a huge variety of H1N1/N2 and H3N2 IAV subtypes in swine worldwide, and it has been established that different lineages are circulating in various regions of the Earth. A common characteristic of swine IAV ecology is the periodic spread of seasonal human viruses. The introduction of seasonal H1N1 and H3N2 IAVs from humans to swine at different times and in various geographical areas has led to the establishment of novel IAV lineages in swine hosts and contributed to the genetic and antigenic diversity of influenza observed in swine [27,44,45,46]. Figure 3 presents the geographical distribution of swine influenza viruses worldwide.

Swine surveillance levels have increased worldwide following the emergence of the pandemic H1N1 virus (H1N1pdm09), as well as still-detectable IAV and/or novel gene lineages not previously recognized in swine, in regions that are not IAV-endemic [22,25,47,48].

### 3.1. Circulation of Swine Influenza Viruses in North America

SI was first identified as a swine respiratory infection in the U.S. in 1918, coinciding with the Spanish human influenza pandemic [49,50]. IAV was first isolated from swine in 1930 by Schope, who demonstrated that the virus caused a respiratory illness similar to human influenza in swine [51]. A phylogenetic analysis of this virus, classical H1N1 (cH1N1) SIV, showed that the cH1N1 lineage was closely related to the 1918 H1N1 Spanish IVs [52] and other isolated human IAVs after the discovery of the SI virus (SIVs) in the 1930s. The cH1N1 virus circulated among swine in the U.S. for almost 80 years with minimal genetic changes [40]. However, in the late 1990s, a triple-reassortant IAV was discovered. It contained the HA, NA, and PB1 genes derived from seasonal human H3N2; PB2 and PA from avian IAV; and NP, M, and NS from IAV cH1N1 of swine [53].

Several outbreaks of respiratory diseases occurred in 1998 among swine herds in North Carolina, Texas, Minnesota, and Iowa. The disease was caused by H3N2 IAVs. Molecular genetic analyses showed that the origin of these SIVs appeared in two diverse ways. The isolate from North Carolina was a product of genetic reassortment, and it contained segments of the IAV H3N2 gene of human origin (PB1, HA, and NA) circulating in 1995 and segments of genes from the cH1N1 porcine lineage (PB2, PA, NP, M, and NS). The strains isolated in Minnesota, Iowa, and Texas constituted the triple reassortants and contained segments of the cH1N1 SIV gene (NP, M, and NS), the same segments of the human virus gene (PB1, HA, NA), and viruses of avian origin (PB2 and PA) [54]. This swine IAV genomic composition is known as the triple-reassortant internal gene (TRIG) cassette [25,55,56,57].

At the end of the 1990s in the United States, in the swine population, in contrast to double-reassortant viruses that did not take root, active circulation of triple-reassortant viruses was observed [58]. Since H3N2 IAVs were almost non-existent among the U.S. swine population, a question arose as to the in-depth study of the virus’s spread. For this purpose, 4382 swine serums collected from 23 states were serologically analyzed using the hemagglutination inhibition (HI) assay [58]. It was established that 28.3% of these animals were exposed to the cH1N1 virus, while 20.5% were exposed to the triple-reassortant-like H3N2 viruses. The HI assay results suggested that viruses antigenically related to the double-reassortment H3N2 virus have not become widespread among the U.S. swine population. In contrast, serodiagnosis of swine serum samples, as well as additional molecular sequencing analysis of six 1999 isolates that had a triple-reassortant genotype, suggested that the H3N2 viruses carrying the avian PA and PB2 genes had spread throughout most of the United States [58].

The presence of avian polymerase genes (PB2 and PA) in the triple-reassortant virus provided the majority of the diversity between the two viruses. The epidemiology of IAVs in swine from the U.S. drastically changed after 1998, when the triple-reassortant H3N2 viruses containing gene segments from the cH1N1 virus (NP, M, and NS), seasonal H3N2 human IAV subtype (PB1, HA, and NA), and avian IAV (PB2 and RA) successfully emerged in the swine population [54,58].

Since 1998, human H3N2 IAVs have been introduced into swine populations at least three times [59,60], resulting in the formation of phylogenetic clusters I, II, and III [61]. Cluster III (C-III) viruses actively spread in North America [62] and then evolved into variants of cluster IV (C-IV) over time [63], which contributed to the conservation and expansion of H3 IAV C-IV [64].

H3N2 viruses subsequently formed reassortants with swine H1N1 IAVs, acquiring the H1N1 or H1N2 subtypes [60,65]. Most fully characterized swine viruses contain the TRIG cassette regardless of their subtype. Reassortant H1 TRIG viruses, having become endemic, therefore continued to circulate in most swine-producing regions of the U.S. and Canada, including other drifting H3N2 variants [58,59,60,63,66], H1N2 [67], and reassortant IV rH1N1 [60,68,69,70,71].

Reassortments observed from 2000 to 2009 are associated with the HA and/or NA protein segments while maintaining the TRIG constellation, which comprised swine (M, NP, and NS gene segments), avian (PB2 and PA gene segments), and human IVs (PB1) [55,69,72,73,74].

The H3N1 viruses were sometimes recognized as bounded outbreaks but were not extensively circulating [75,76]. Furthermore, in 2006, TRIG was demonstrated to have taken the avian HA H2 and N3 variants, resulting in a new triple-reassortant H2N3 SIVs [77]. Yassine et al. [78] showed that North American swine TRIG-containing viruses can infect turkeys, which plays a certain role in IAV epidemiology in swine hosts and human hosts. Several authors reported that genetically related TRIG-containing swine viruses were also detected in Korea, Vietnam, and China [79,80,81].

There are reports on the introduction of IAV with the HA H1 gene of human H1N2 (hu-like H1) seasonal viruses, which are both molecularly and genetically different from the SIVs cH1N1 in Canada [82]. Since 2005, TRIG-containing hu-like viruses H1N1 and H1N2 have appeared in U.S. swine populations [83]. However, their TRIG genes are similar to those found in contemporary triple-reassortant SI viruses.

To better represent the evolution of H1 IAV circulating in North America, a cluster classification was proposed. cH1N1 IAVs, circulating among swine since 1918, evolved, based on the genetic composition of the HA gene, into contemporary a-, b-, and c-clusters, while strains with the H1 HA subtype most similar to seasonal human H1 IAVs form a d-cluster [83]. All four HA gene clusters (a, b, c, and d) are found with the NA genes of the N1 or N2 subtype. The HA genes from the d-cluster of viruses presumably originated from two different introductions of human seasonal viruses, with HA from the H1N2 and H1N1 viruses phylogenetically differentiated into two subclusters of swine origin, d1 and d2, respectively [69,84]. In strains isolated in the U.S. during 2008–2010, the d-cluster HA genes paired with either the N1 or the N2 IAV gene of human origin [85,86]. Until 2009, the H1 IAV evolved as a result of the reassortant formation while maintaining TRIG as the basis and generating genetically and antigenically different viruses [25,69]. Since then, H1N1pdm09 has become firmly established in the U.S. swine population with the emergence of subsequent second-generation reassortants [87,88,89].

A(H1N1)pdm09 IV has also become established in swine in North Carolina and co-circulates with previously enzootic SI virus strains, including avian H1N1 and human-like H1N2 [90]. In 2010, a reassortant IAV (H1N2r) was isolated that caused mild clinical disease in swine in North Carolina. This reassortant virus has a gene constellation, including the internal gene cassette of the A(H1N1)pdm09 viruses and HA and NA genes of the swine-origin H1N2 virus. This reassortant virus has been shown to have both zoonotic and reverse zoonotic potential and no apparent increased virulence or transmissibility compared to A(H1N1)pdm09 viruses [91,92].

During the swine influenza surveillance in 2012, a reassortant IAV strain containing HA and NA from human seasonal IAV was detected. These viruses subsequently reassorted, maintaining only the human-origin H3, which resulted in a novel lineage of viruses that became the most frequently revealed H3 branch in U.S. swine (2010.1 HA clade) [64,93,94]. Powell et al. [95] found that compared to the 2010.1 viruses from 2012 and 2014, the 2010.1 strains from the 2015–2017 period led to equivalent macroscopic lung lesions in swine. A single mutation in amino acid residue 145 in a previously identified HA antigenic motif was associated with a change in antigenic phenotype, potentially reducing vaccine effectiveness. The 2010.1 viruses circulating in swine after 2012 significantly differed from both the H3N2 viruses that existed prior to 2012 in swine and human seasonal influenza H3N2 viruses and showed continued evolution within this lineage [95].

In 2017, the Iowa State University Veterinary Diagnostic Laboratory detected a reverse zoonotic transmission of seasonal human IAV to swine in Oklahoma. A pairwise comparison between the human H3 IAV (H3.2010.2) HA sequences detected in swine and the most similar 2016–2017 seasonal human H3 sequences revealed 99.9% nucleotide identity [96]. H3.2010.2 is a phylogenetic clade of H3N2 circulating in swine. This clade became established following the spillover of a seasonal human H3N2 virus during the 2016–2017 influenza season. H3.2010.2 transmitted and adapted to the swine host and demonstrated reassortment with the internal genes from strains endemic to swine but maintained the human HA and NA. It is genetically and antigenically distinct from H3.2010.1 H3N2, detected earlier in 2010. It was shown that seasonal human-to-swine transmission of IAV became established in the population through adaptation and contributed to the genetic and antigenic diversity of IAV circulating in swine [96].

Exposure to IAVs and infectious individual swine assists in the introduction, transmission, and spread of diverse IAVs. Influenza strains circulating among swine can cause outbreaks in humans. In October 2020, a swine-origin H1N2 IAV variant was detected in a person in Alberta, Canada [97]. The source of the infection was a local swine farm, where a contact person worked in the household. A phylogenetic analysis showed that the isolate was very similar to the strains found on this farm in 2017.

Agricultural fairs also bring humans into close contact with swine and provide a unique interface for the zoonotic transmission of IAVs. Agricultural fairs are held every summer in the United States, where people are exposed to genetically diverse IAVs circulating among exhibition swine. As a result of such fairs, more than 450 laboratory-confirmed cases of zoonotic infections have been recorded since 2010 [98].

An understanding of the IAV transmission dynamics through exhibition swine is therefore critical to reducing the high incidence of various IAV variants registered in connection with agricultural fairs.

### 3.2. Circulation of Swine Influenza Viruses in South America

IAVs have been found in South American swine populations. Several IAV lineages are circulating among swine populations in Latin America, which apparently existed but remained undetected for many years; however, these lineages were established after the emergence of the seasonal human virus and the global movement of swine. The emergence of IAV with H1 HA from the classical clade of swine lineage has been reported in Mexico, including viruses with HA that share a common ancestor with the H1N1pdm2009 virus, as well as viruses with HA and NA belonging to the H1N1 lineage of Eurasian swine [99,100,101]. Two lineages of H3N2 IAVs were also detected in Mexico: one probably resulted from C-IV H3 importation from North America, and the second resulted from a unique seasonal introduction to humans since the mid-1990s [99].

A report was received from Argentina in 2009 in which clinical disease and the isolation of H1N1pdm IAV were recorded on a commercial swine farm. Later, in 2011, the reassortant H1N1, H1N2, and H3N2 IAVs with internal genes from the H1N1pdm virus were detected. Clinical, pathological, and virological evidence suggested that IV infection spread widely among swine farms in Argentina [102,103,104,105,106].

Since the emergence of H1N1pdm in 2009, IAV outbreaks have also been detected in Brazilian swine herds [99,100,107,108,109,110,111,112,113,114,115,116,117]. A total of 107 strains of H1N1, H1N2, and H3N2 IAVs were characterized during the surveillance of swine in Brazil from 2011 to 2020 [118,119]. A phylogenetic analysis based on surface protein segments (HA and NA) showed that since the mid-1980s, seasonal human IAVs have been introduced at least eight times into the swine population in Brazil. The analysis identified three H1 genetic clades within the 1B lineage and an H3 lineage that has diversified into three genetic clades. The N2 segment from seasonal human H1N2 and H3N2 IAVs was detected six times in swine, and the N1 segment from the human H1N1 virus was also identified once. An additional analysis revealed further reassortment with the H1N1pdm09 viruses, which confirmed the significant contribution of human IAVs to the genetic diversity of IAV in swine and the need for IAV surveillance in swine [118].

Swine H1N2 IAVs, which co-circulate with A(H1N1)pdm09-like viruses, have been recognized in Chile [120,121]. A genetic analysis based on the HA1 domain and an antigenic analysis by the HI assay demonstrated the presence of three antigenic clusters: Chilean H1A (ChH1A), Chilean H1B (ChH1B), and A(H1N1)pdm09-like. It was established that the antigenic sites of the ChH1A and ChH1B strains differed from those of commercial vaccine strains by 10–60% at the amino acid sequence level. These findings confirmed the importance of antigenic analyses and genetic testing to evaluate control measures such as vaccination. These results emphasized the need for ongoing monitoring to determine further transmission of antigenic variants in Chilean swine populations [99,122,123].

### 3.3. Circulation of Swine Influenza Viruses in Europe

SIV is one of the most significant primary causative agents of swine respiratory diseases in Europe. Three subtypes of IAVs, H1N1, H1N2, and H3N2, have been circulating in the European swine population for decades. These viruses remain endemic in the European swine populations, but they differ in antigenic characteristics from viruses circulating among swine elsewhere.

Since the mid-twentieth century, cH1N1 IAVs have been widely spread in European swine populations and North American swine herds. However, in the late 1970s, cH1N1 viruses were replaced by avian H1avN1 IAVs, which were transmitted from wild ducks to swine and later spread to Asia [124]. In early 1979, the emergence of avian H1avN1 IAVs was reported in Belgium [124] and, later, in Germany and France [125,126,127]. Subsequently, during a long-term 13-year SI surveillance in Germany, the persistent spread of the Eurasian H1avN1 virus was established, as well as the emergence of a novel H1N1 reassortant with human HA and avian NA genes. There has also been a report of a zoonotic infection caused by H1avN1 1C.2.2 IAV and detected in a German child [128]. In addition, the evolution of the A(H1N1)pdm09 antigenic drift variant has been observed [129].

In Serbia, in 2016–2018, during the monitoring of IAV circulation on 13 swine farms, 255 samples were obtained from animals. The IAV genome was detected in 24 samples. As a result of virological studies, eight strains of IAV were isolated. Sequence studies of the HA and NA genes revealed the circulation of H1N1 and H3N2 IAVs. IAVs with the subtype H1 were classified as 1C.2.1 and 1A.3.3.2. clades. The nucleotide sequences of the genes HA H1 showed that three viruses belong to the H1N1pdm09 lineage, and four belong to the Eurasian “avian-like” lineage H1avN1. Based on their NA gene sequences, these seven viruses belong to the Eurasian avian lineage H1avN1. The HA and NA genes of the H3N2 subtype virus belong to the A/swine/Gent/1/1984-like H3N2 lineage [130,131,132].

The genetic diversity of SIVs was investigated in northern Italy from 2017 to 2020. High genotypic diversity was found among the H1N1 and H1N2 strains, while the H3N2 strains showed a stable genetic pattern. The data obtained confirmed the need for continuous monitoring, since the evolution of IAVs could result in the emergence of isolates with zoonotic potential [133].

H1avN1 IAV has therefore become the dominant lineage on both continents and played a key role in the present-day ecology of endemic viruses, both in these regions and worldwide [134].

The European swine influenza H3N2 viruses of human lineage (A/Hong Kong/1/68) emerged in the 1980s, and they differed from the contemporary H3N2 viruses in North America [40,135,136,137]. They acquired the internal H1avN1 IAV gene cassette as a result of reassortment and had two surface protein genes (HA and NA) from the 1968 Hong Kong IAV and six internal genes (M, PB1, PB2, PA, NP, and NS) from an endemic avian H1N1 virus [138,139]. However, this virus rapidly disappeared from the European swine population and, in 1984, was replaced by another virus with a very similar genetic composition [140]. It quickly spread in the population of European swine. Epizootics have been recorded in Belgium [139,141], France [142], Germany [143,144], England [145], Italy [135,138], Spain [146,147], and the Netherlands [148,149].

H1N2 IAVs, first isolated in the UK, became widespread among European swine in the mid-1990s [150,151,152]. Swine human-like H1N2 (H1huN2) IAVs retained the genotype of reassortant H3N2 viruses but acquired the H1 gene from the seasonal human H1N1 virus of the 1980s. H1huN2 IAVs later spread in France (1997 and 2000–2018), Italy (1998), Belgium and the Netherlands (1999 and 2014–2019), Germany (2000, 2009, 2014–2015), and Denmark (2011–2014) [129,151,152,153,154,155,156,157,158,159,160,161,162,163,164,165,166].

These three subtypes (H1avN1, H3N2, and H1huN2) therefore share internal genes but have clearly distinguishable HAs. All three IAV subtypes became established in the European swine populations and gradually replaced previously circulating cH1N1 IAV [48,134].

H1N1pdm09 IAV has also been prevalent in European swine since the 2009 pandemic [167,168,169,170]. The first reports of H1N1pdm09 IAV came from England and Northern Ireland in 2009 [167]. H1N1pdm09 IAVs were later detected in Finland [171,172] and Germany [169]. A swine reassortant, H1N2r IAV, was isolated in 2010, which caused mild clinical disease in swine in the United Kingdom. This reassortant virus had a novel gene constellation, including the internal gene cassette of the H1N1pdm09 viruses and HA and NA genes from swine-origin H1N2 [92]. In 2016, reports came from Greece about the detection of H1N1pdm09, H1N1, H1N2, and H3N2 IAVs in swine serums collected during 2002–2004 and 2010–2012 [173]. Serological surveillance in Norway from 2009 to 2017 also detected the circulation of H1N1pdm09, H1N1, H1N2, and H3N2 IAVs [174]. Data on the zoonotic transmission of H1N1pdm09 IAV from swine to humans appeared in Germany in 2009–2010 [175] and in France in 2018 [170].

As elsewhere, the introduction of H1N1pdm09 into European swine resulted in an increase in second-generation reassortants between human H1N1pdm09 and established endemic lineages [176,177,178,179]. The circulation of reassortant viruses was reported in the UK [180], Italy [181,182], Germany [157,169,175], France [183], Sweden [184], and Denmark [160,185]. In addition, certain unusual subtypes, such as H3N1 and H1N7 viruses, have been revealed in European swine, but they were apparently unable to survive and spread among swine [186,187,188].

The emergence of H1N1pdm09 and reassortment with endemic swine IAVs therefore significantly diversified the swine IAV ecology in Europe [175,180,181].

### 3.4. Circulation of Swine Influenza Viruses in Asia

SIV demonstrates a high level of diversity in Asian swine populations due to the introduction of viruses from Europe and North America [81,189]. It has been established that the first isolation of IAVs in Asia was in 1974 (swine/Hong Kong/1/74), although the first detection of cH1N1 was recorded in Chinese swine during the Spanish flu pandemic in 1918. SIV observations conducted in Hong Kong and Japan since the mid-1970s have shown that cH1N1 is widely spread in numerous Asian regions and countries [190,191]. cH1N1s have been discovered in Hong Kong, Japan, India, Taiwan, Singapore, Iran, Thailand, Korea, Vietnam, and Malaysia [181,192,193,194,195,196,197,198,199,200,201,202].

The stock of IAV genetic diversity in swine herds from China, South Korea, Vietnam, and other Asian countries has increased due to the intercontinental movement of swine and their viruses [45,203]. This led to the spread of the European H3N2 and H1avN1 viruses, along with North American TRIG viruses introduced in the late 1990s to early 2000s.

The findings of evolutionary studies have shown that cH1N1 SIV and the 1918 pandemic influenza H1N1 virus had a general ancestor or were very closely related [204,205,206]. The relationship between human and swine H1N1 viruses may be similar to that between human and H1N1pdm09 viruses. Since its appearance, H1N1pdm09 has been constantly transmitted from humans to swine [102,207]. In addition, the sporadic isolation of human-like H1N1 viruses has been observed in swine in Japan [208] and, later, in China [209,210].

The H1N1pdm09 virus was introduced to swine and reassorted with the endemic subtypes in China [57], Japan [211,212], and Thailand [213]. In many Asian countries, H1N1, H1N2, and H3N2 viruses included different internal gene segments from the H1N1pdm09 virus [57,195,214,215,216,217,218,219,220,221,222,223,224,225,226,227], resulting in numerous highly complex reassortant viruses [81,217,225,227,228,229].

Avian influenza H1avN1viruses were detected along with cH1N1 in clinically healthy swine in Southern China in 1993 [198,215]. Later, in 2001, H1avN1 viruses that caused the avian influenza Eurasian variant were detected in swine in Hong Kong and Thailand [45,230].

In Asia, the circulation of reassortant H1N1 swine influenza viruses of North American and/or European origin has been recorded among swine. Reports of H1N1 swine reassortant virus circulation came from Korea [79,231], Thailand [230,232], and Malaysia [233]. There is serological evidence for the prevalence of H1N1 SIVs in Cambodian swine in 2006–2010 [234]. H1N1 SIVs were recorded in Bhutan in 2011–2012 [235]. H1N1 SIVs were detected in Russia in 2016 [236]. A genetic analysis of the H1N1 virus isolate revealed 90% identity similarity between HA and NA genes and those found in U.S. humans in the 1980s.

The first outbreak of influenza in swine caused by reassortant H1N2 viruses determined from the human-like variant of swine H3N2 and cH1N1s viruses was observed in Japan in 1978; later, outbreaks were recorded during 1989–1990 [237].

These viruses, endemic in Japanese swine populations, established a genetically stable lineage [238]. A similar reassortant H1N2 IAV gene cassette was detected in swine in Taiwan [239], China [45,240], and India [241].

Since 2002, a genetically stable line of North American reassortant H1N2 viruses containing HA cH1N1s has become widespread in Korea [79].

Since the 1970s, in Asia, the H3N2 influenza virus has been transmitted from humans to swine on multiple occasions, and the first report of the virus being isolated from swine in Taiwan came a year after the human pandemic in Hong Kong [242]. Outbreaks of viral infection caused by H3N2 IAVs in swine have also been reported in Hong Kong, Japan, Korea, India, and Thailand [189,243,244,245,246,247,248,249,250], but these H3N2 viruses could not become established in swine herds [214,251].

In the 1980s, swine H3N2 viruses were isolated in China, containing human-like HA and NA and the cH1N1 internal gene segments. At about the same time, two triple-reassortant H3N2 viruses were isolated from swine in China that were not genetically related to the North American or European reassortant viruses. In 1999, SIVs appeared in China: the European reassortants of human-like H3N2 SIVs. Further reassortment of these viruses with circulating cH1N1 influenza viruses subsequently resulted in triple-reassortant strains [214]. In addition, the European reassortant human-like H3N2 SIVs were genetically reassorted with circulating human H1 or H3 strains in Thailand [230,232]. The circulation of European viruses in swine was confirmed by serological studies in Malaysia [233]. Since 1998, SIV strains have been isolated in Korea, which are triple-reassortant H3N2 IAVs of the North American swine lineage with three different human-like HAs [79,252]. The swine showed signs of respiratory disease, but no deaths were recorded. There are also several reports on the prevalence of the H3N2 virus in swine in Sri Lanka during 2004–2005 [253], in Laos during 2009 [254], and in Cambodia during 2011–2012 [255].

Influenza epizootics among swine represent a serious problem in the Republic of Kazakhstan. In 1984, due to a virological examination of piglets with clinical signs of respiratory diseases on swine farms situated in East Kazakhstan, three strains of the A/H1N1 influenza virus were isolated [256]. Due to the nature of the interaction with a panel of monoclonal antibodies, the Kazakhstan isolates showed similarities with the A/England/333/80 (H1N1) virus. The performed immunological analysis made it possible to classify the strains isolated from swine as drift variants of the human influenza A(H1N1) virus.

Reassortant A/H3N6 strains were also isolated from swine in Kazakhstan in 1986. The viruses of this complex were characterized by significant antigenic heterogeneity. The populations of several strains contained virions whose enzymatic activity was suppressed by immune serum against NA N6 [256].

In 2008–2009, nine isolates collected from uneven-aged swine belonging to peasant farms of the Republic of Kazakhstan were identified, of which four had A(H1N1), one had A(HswN1), and four had the A/H3N2 antigenic formula. A serological analysis performed to retrospectively confirm the etiological role of influenza viruses showed the presence of antibodies against influenza A/H1N1, A/HswN1, and A/H3N2 viruses in animals, which completely coincided with the results obtained from the isolation of viruses in chicken embryos [256].

In 2013–2014, a serological analysis of 492 blood serums collected from swine belonging to peasant farms in various regions of Kazakhstan was conducted. Data obtained in the study indicated the co-circulation of influenza A/H1N1 and A/H3N2 viruses among animals during the specified period [256].

There are also data on the circulation of H1N1 and H3N2 SIVs among the swine population in Kazakhstan during 2017–2022 [256,257,258,259].

A genetic analysis of the H1N1 IAV strain, isolated in Kazakhstan in 2020, showed that it belongs to clade 1A.3.2.2, lineage 1A. This clade has a wide global distribution, which includes the 2009 H1N1 pandemic strains [18]. The findings emphasized the importance of ongoing SIV monitoring to identify the possibility of the cross-species transmission of this infectious agent.

Thus, the IVs that infect swine most frequently belong to the H1N1 subtype, but at the same time, the H1N2, H3N1, and H3N2 variants are circulating among them. Sometimes, swine are infected with more than one virus simultaneously, resulting in a reassortant strain containing genes from different sources. Reassortment plays a significant role in the emergence of novel virus variants, particularly in the origin of pandemic strains.

### 3.5. Circulation of Swine Influenza Viruses in Africa and Australia

H1N1pdm09, H1N1, and H3N2 IAVs have been reported in various African countries [260,261], such as Egypt [262,263], Nigeria [264,265,266,267,268], Ghana [269,270], Cameroon [271,272,273], Kenya [274,275,276], Réunion Island [277], Uganda [278], Togo [279], and Burkina Faso [280]. In addition to H1N1pdm09, the circulation of avian influenza viruses such as H5N1, H5N2, and H9N2 has been recorded in Nigeria and Egypt [262,267].

Swine influenza in Australia was first reported in 2009, when a herd of 300 sows was infected with the H1N1pdm09 virus. The outbreak occurred as a result of human-to-swine virus transmission [281,282]. The outbreaks of influenza H1N1pdm were detected on three swine farms in three different states of Australia. Further analysis of the outbreak in Queensland resulted in the identification of two distinct IAV strains in swine. Two employees working in the same piggery were also infected with the same two strains found in swine. Although no reassortment was identified in the above cases, the ability of these viruses to be transmitted between swine and humans was evident, emphasizing the importance of monitoring swine to identify novel influenza infections [282].

An outbreak of SI occurred in Western Australia in 2012 [283], and the infection spread among both swine and farm workers. A molecular analysis of the isolates revealed the circulation of H1N2, H3N2, and H1N1pdm09 IAVs. H1N2, H3N2, and H1N1 SIVs later continued to circulate in Western Australia and Southern Queensland right up to 2016 [284]. A public health investigation identified an unrecognized zoonotic risk and the potential threat of a pandemic in the future [283].

In 2018, there was a report of a 15-year-old teenage girl from Australia infected with A(H3N2) SIV. The virus contained the HA and NA genes derived from the 1990s-like seasonal human viruses and internal protein genes from H1N1pdm09 IAV, emphasizing the potential risk to human health in Australia [285].

## 4. Circulation of Avian Influenza Viruses in Swine

H1N1 and H3N2 IAVs mainly circulate among swine populations in North America, Europe, and Asia. However, there have been no cases of transmission of the 1957 Asian pandemic H2N2 virus to swine. There is also cross-species transmission of avian viruses to swine on these continents. The reassortant SIVs presently circulating have at least one component of the avian virus gene. Although natural infections of swine with avian influenza viruses have been reported in numerous parts of the world, only the European avian H1avN1 virus has adapted to swine [166,286,287,288]. Other subtypes of avian influenza virus have been found sporadically in swine in North America and Asia. There are reports of sporadic isolation of atypical H1N7 and H3N1 IAVs from swine in Europe [186,287] and the H4N6, H3N3, H3N1, and H2N3 subtypes in North America [75,77,289]. Numerous interspecies transmissions of H9N2 viruses from host birds to swine occurred in Asia [290]. H9N2 IAVs isolated from swine in Hong Kong in 1998 were fully avian IAV variants [249,290,291]. However, since 2003, reassortant avian strains have emerged with certain genes closely associated with the highly pathogenic avian influenza (HPAI) H5N1virus [292,293,294,295,296]. Infection with the avian H5N1 virus was also detected in swine in Indonesia [297] and Vietnam [231]. The avian H5N2 virus has been transmitted from wild migratory birds to Korean swine [298].

H3N1 SIV, which is a reassortant virus with genes of the human-like H3N2 isolate and circulating cH1N1SIVs, has been isolated from swine in Taiwan [239]. There are data on the circulation of H3N1 SIVs in Indonesia and Korea [299]. H3N1 SIVs circulating in Korea represented a reassortment of co-circulating North American reassortant H1N1 and H3N2 viruses [299].

Equine-like H3N8 SIVs were also isolated from swine in Central China [300].

Thus, some avian IAVs, such as H9N2, H5N1, H5N2, H4N8, and H6N6, as well as equine H3N8 IAV, have been sporadically detected in swine from certain Asian countries [81,189,300,301,302]. The simultaneous circulation of so many genetically diverse IAVs in swine explains the very complex ecology of swine IAVs in Asia.

## 5. Conclusions

Swine play an important role in the formation of present-day influenza ecology. Swine have specific receptors for avian, porcine, and human IAV in the tracheal epithelium, which may contribute to the emergence of new reassortant variants that could potentially pose a public health threat [8,249,303]. The complex interaction of IAVs of human, avian, and porcine evolutionary origin in swine is supported by the detection of multiple IAV subtypes in swine populations, including H1, H2, H3, H4, H5, H7, and H9 [304]. Swine play an important role in the epidemiology of IV, which is dangerous to humans [305]. For example, influenza A(H1N1)pdm09 is thought to have been present in swine herds for several months before it became a pandemic strain in humans [35].

Human-to-swine transmission of IV occurs much more frequently than swine-to-human transmission [30]. Despite the genetic diversity of SIVs found in the swine population, only H1 and H3 IAVs formed stable lineages similar to human IVs. The nature of H1 and H3 viruses nevertheless differs between the two host populations. In addition, the distribution of subtypes and genotypes of endemic IAVs varies greatly by geographic region due to the repeated introduction of avian and human influenza viruses. An exception is H1N1pdm09, which is mainly widespread due to multiple human-to-swine reintroductions [57,102,167,176,306,307,308,309,310,311,312]. New cases of infection with avian influenza H5N1 virus have been confirmed in dairy cattle and pigs [313,314]. However, no information has been found on the presence of swine-origin IVs in dairy cows.

The continuous monitoring and genome-wide genetic characterization of SIVs make it possible to obtain a complete picture of the infectious disease process, predict the epidemiological and epizootic situation, and choose the right strategy and tactics for preventive and anti-epidemic measures. Understanding the transmission modes is of decisive importance for developing effective control strategies and making reasonable surveillance recommendations.

This study may provide the information needed to develop a more effective method for monitoring SIVs and selecting countermeasures to prevent interspecies transmission, which could become a potential pre-pandemic situation.

## Data Availability

Not applicable.
